# Spatial access to restaurants and grocery stores in relation to frequency of home cooking

**DOI:** 10.1186/s12966-017-0640-6

**Published:** 2018-01-16

**Authors:** Maria Gabriela M. Pinho, Joreintje D. Mackenbach, Hélène Charreire, Jean-Michel Oppert, Helga Bárdos, Harry Rutter, Sofie Compernolle, Joline W. J. Beulens, Johannes Brug, Jeroen Lakerveld

**Affiliations:** 1 Department of Epidemiology and Biostatistics, Amsterdam Public Health research institute, VU University Medical Center Amsterdam, Amsterdam, The Netherlands; 2 Equipe de Recherche en Epidémiologie Nutritionnelle (EREN), Centre de Recherche en Epidémiologie et Statistiques, Inserm (U1153), Inra (U1125), Cnam, COMUE Sorbonne Paris Cité, Université Paris 13, Bobigny, France; 3 Université Paris Est, Créteil, UPEC, Lab-Urba, Créteil, France; 4 Sorbonne Universités, Université Pierre et Marie Curie, Université Paris 06; Institute of Cardiometabolism and Nutrition, Department of Nutrition, Pitié-Salpêtrière Hospital, 47-83 Boulevard de l’Hôpital, 75013 Paris, France; 5 Department of Preventive Medicine, Faculty of Public Health, University of Debrecen, Debrecen, Hungary; 6 ECOHOST – The Centre for Health and Social Change, London School of Hygiene and Tropical Medicine, London, UK; 7 Department of Movement and Sport Sciences, Faculty of Medicine and Health Sciences, Ghent University, Ghent, Belgium; 8 Julius Center for Health Sciences and Primary Care, University Medical Center Utrecht, Utrecht, The Netherlands; 9 Amsterdam School of Communication Research (ASCoR), University of Amsterdam, Amsterdam, The Netherlands

**Keywords:** Home cooking, Food environment, Restaurants, Grocery stores, Spatial analysis, Adults

## Abstract

**Background:**

Little is known about the relation between the neighbourhood food environment and home cooking. We explored the independent and combined associations between residential neighbourhood spatial access to restaurants and grocery stores with home cooking in European adults.

**Methods:**

Data of 5076 participants of the SPOTLIGHT study were collected across five European countries in 2014. Food retailers were classified into grocery stores (supermarkets and local food shops) and restaurants (full-service restaurants, fast food and take-away restaurants, café/bars). We used multinomial logistic regression models to test the associations between tertiles of spatial access to restaurants and spatial to access grocery stores and the outcome ‘frequency of home cooking’ categorized into 0-3; 4-5; and 6-7 days/week. Additive interaction analysis was used to test the combined association between access to grocery stores and to restaurants with home cooking.

**Results:**

Mean age was 52.3 years; most participants were women (55.5%) and completed higher education (53.8%). Residents with highest access to restaurants had a reduced likelihood of home cooking 6-7 days/week (vs. 0-3 days/week) (relative risk ratio (RRR) 0.42; 95%CI = 0.23-0.76) when compared with lowest access to restaurants. No association was found for spatial access to grocery stores. Additive interaction analysis showed that individuals with medium access to grocery stores and highest access to restaurants had the lowest likelihood (RRR = 0.29, 95%CI = 0.10-0.84) of cooking 6-7 days/week when compared to individuals with lowest access to restaurants and highest access to grocery stores.

**Conclusion:**

Greater neighbourhood spatial access to restaurants was associated with lower frequency of home cooking, largely independent of access to grocery stores.

**Electronic supplementary material:**

The online version of this article (10.1186/s12966-017-0640-6) contains supplementary material, which is available to authorized users.

## Background

The food environment is considered an important ‘upstream’ determinant of dietary habits, including home cooking [1–3]. The presence and type of food stores in a neighbourhood may influence dietary habits in different ways. For instance, while the neighbourhood presence of grocery stores and farmers’ markets has been associated with healthier diets [4–6], the presence of convenience stores, takeaway and fast food outlets has been linked to less healthy food consumption [4, 7, 8]. However, evidence for the influence of food retailers on dietary habits is mixed, and there is a need to explore new definitions of access to the food environments [9]. The presence of food retailers cannot be isolated from their broader food environment; e.g. supermarkets may provide opportunities for buying ingredients for healthy meals, but the extent to which this is actually done may be influenced by the availability of takeaway or ready meal options. Few studies to date have taken the broader food environment into account in this context. For example, Burgoine et al. (2014) demonstrated that takeaway food outlets exposure was only associated with takeaway meal consumption and obesity when adjusted for exposure to supermarkets [8].

In recent decades, many people in middle and high-income countries have adopted diets with more highly processed foods, including ready-to-eat or takeaway meals, and fewer home cooked meals prepared from fresh ingredients [10, 11]. Promoting home cooking might contribute to healthier diets, as cooking at home has been associated with less consumption of fat and sugar [12], more fruit and vegetables [4], higher quality of diet, better adherence to dietary guidelines and lower adiposity [13, 14]. A recent systematic review of the determinants and outcomes of home cooking found that the majority of available studies explored only individual level determinants of cooking [15, 16]. Despite considerable knowledge about the individual correlates of home cooking [17–20], no study so far has investigated if and how the food environment is associated with home cooking.

We aimed to investigate the association of residential neighbourhood spatial access to grocery stores (supermarkets and local food shops) and to restaurants (full-service restaurants, fast food and take-away restaurants, café/bars) with frequency of home cooking. While statistical adjustment for other food retailers can reveal independent effects of a single food retailer with dietary habits, it may be that there are joint effects as well. Therefore, a second aim was to investigate the combined association between access to grocery stores, and to restaurants, on home cooking by testing additive interaction. We hypothesized that greater spatial access to grocery stores is both independently and jointly associated with higher frequency of home cooking and greater spatial access to restaurants is both independently and jointly associated with lower frequency of home cooking.

## Methods

### Study design, sampling and participants

This study was part of the SPOTLIGHT project. SPOTLIGHT focused on obesity prevention, particularly through the identification of the determinants of obesity and obesogenic behaviours [21]. Within the project, an online cross-sectional survey was conducted in five urban regions across Europe: Ghent and suburbs (Belgium), Paris and inner suburbs (France), Budapest and suburbs (Hungary), the Randstad region (the Netherlands) and Greater London (UK). Neighbourhoods were sampled based on a combination of residential density and socioeconomic status (SES) data at the neighbourhood level. This resulted in four types of pre-specified neighbourhood types: low SES/low residential density, low SES/high residential density, high SES/low residential density and high SES/high residential density. In each country, three neighbourhoods of each type were randomly sampled (i.e. 12 neighbourhoods per country, 60 neighbourhoods in total). The sampled neighbourhoods were on average smaller in Paris (0.3 km^2^) and largest in Greater London (3.6 km^2^). The neighbourhood population across the five urban regions was, on average, 2700 adults per neighbourhoods. The country specific lowest population was found in Ghent and suburbs (946 adults per neighbourhood on average) and the highest was found in Greater London (5607 adults per neighbourhood on average). More detailed information on the sampling, design and participant recruitment have been described elsewhere [22].

Adults (≥18 years) living in the selected neighbourhoods were invited to participate in an online survey that included questions on demographics, neighbourhood perceptions, social environmental factors, health, motivations for and barriers to healthy behaviour, obesity-related behaviours, dietary behaviours, and self-reported weight and height. A total of 6037 (10.8%, out of 55,893) individuals participated in the study between February and September 2014. Local ethics committees in each participating country approved the study, and all survey participants provided informed consent.

### Measures

#### Spatial access to grocery stores and spatial access to restaurants (independent variables)

Using the validated SPOTLIGHT Virtual Audit Tool (SPOTLIGHT-VAT), objective food environment data were obtained in 58 residential neighbourhoods. The SPOTLIGHT Virtual Audit Tool allows the researcher to virtually walk through a neighbourhood using Google Street View and assess features of the built environment by pinpoint locations of interest. All streets that were captured by Google Street View in the selected neighbourhoods were virtually audited. A total of 42 items representing 8 dimensions of the food and physical activity environment were identified and geo-localized at the address level in a GIS [23]. The dimensions of the food environment used in this study were based on the work of Lake et al. (2010), who defined food retailers such as restaurants, fast food outlets and café/bars as places that mostly sell meals to be eaten away from home, while supermarket are places that mostly sell ingredients to be prepared at home [24]. We then considered supermarkets and local food shops as ‘grocery stores’ and full-service restaurants, fast food and take-away restaurants, and cafés/bars as ‘restaurants’. It was not possible to perform the virtual audit in two neighbourhoods because they were not covered by Google Street View at the time of data collection.

Based on a model first described by Stewart (1941) for application to the food environment [25], we used a measure of spatial accessibility that reflects both distance from the participant home address to each food retailer (proximity) and the total number of food retailers (density) in the individuals’ residential neighbourhood (defined by administrative limits). For each participant, two scores were calculated using ArcGIS version 10.4; one score reflecting their spatial access to grocery stores and another reflecting their spatial access to restaurants. The first step towards obtaining these scores was to calculate the Euclidian distance from individuals’ houses to each grocery store and to each restaurant in their residential neighbourhood. Assuming that the surroundings of the individuals’ neighbourhood might be part of their activity space, and to take the direct environment into account of those who lived close to the administrative neighbourhood boundary, food outlets within 300 m buffer zone around the residential neighbourhoods were also considered. In a next step, each grocery store and restaurant were weighted according to an inverse function of the distance. Finally, the inverse weighted distances to each grocery store and to each restaurant were summed in order to create two spatial access scores, which were then assigned to each individual. Due to the skewed distribution and difficult interpretation in terms of units of access, these scores were split in tertiles reflecting lower, medium and higher spatial access. This resulted in two categorical variables: ‘spatial access to grocery stores’ and ‘spatial access to restaurants’.

#### Frequency of home cooking (outcome variable)

The frequency of cooking at home was assessed by asking participants the following question: *How many days a week do you, or does someone in your household, prepare a meal using ingredients as opposed to eating ready or takeaway meals?* There were eight response options ranging from *less than once a week* to *7 days a week.* Based on the data distribution and previous literature [13, 14], we split the variable into 3 categories: low frequency of home cooking (0 – 3 days per week; *n* = 604); medium frequency of home cooking (4 – 5 days per week, *n* = 977); and high frequency of home cooking (6 – 7 days per week, *n* = 3026).

### Covariates

We collected information on age, sex, height, weight, educational attainment, household composition, employment status, urban regions and perceived barriers to healthy eating. Due to differences between the educational systems across the countries, self-reported educational attainment was categorized into two groups: ‘lower’ (secondary education or less) and ‘higher’ (college or university level). Household composition was categorized into three groups: ‘1 adult, no child’, ‘2 adults, no child’, ‘adult(s) and child(ren)’. Employment status (which includes people who were employed or in education) was classified into two groups: ‘yes’ or ‘no’. Since we have found previously that perceived barriers to healthy eating may influence dietary behaviours and its relations with the food environment [26, 27], we used the variable ‘number of perceived barriers to healthy eating’ - ranging from 0 to 8 – as sensitivity analysis in an additionally adjusted model.

### Statistical analysis

We excluded 961 individuals from the analysis because their residential addresses were located outside the selected neighbourhood or because individuals were living in neighbourhoods not covered by Google Street View at the time of the virtual audit. This resulted in an analytical sample of 5076 participants. Assuming that data were missing at random, we handled missing data by performing multiple imputation to all variables (including outcome) [28]. As the percentage of missing values ranged from 1% (age) to 18.5% (number of perceived barriers to healthy eating), we chose to impute 20 datasets, following the recommendations of Rubin [29] and Bodner [30]. We used the pooled results from the 20 imputed datasets in the multinomial logistic regression models, while descriptive analyses were performed on non-imputed data.

To test the independent association of the two measures of exposure with home cooking, we built three different multinomial logistic regression models with frequency of home cooking as the outcome. Model 1 has ‘spatial access to restaurants’ as independent variable. Model 2 has ‘spatial access to grocery stores’ as independent variable and Model 3 has both ‘spatial access to restaurants’ and ‘spatial access to grocery stores’ as independent variables. All models were adjusted for age (continuous), sex, educational attainment, BMI (continuous), household composition, employment status, and urban region. To test the joint association of spatial access to grocery stores and spatial access to restaurants on the frequency of home cooking, we built a model with an additive interaction term between access to grocery stores and access to restaurants as independent variables. We considered those who hypothetically have the greater likelihood of cooking at home, i.e. those with highest access to grocery stores and lowest access to restaurants as reference category. This model was adjusted for age (continuous), sex, educational attainment, BMI (continuous), household composition, employment status, and urban region. An additional model (Model 3b) was built as sensitivity analysis. This model is like Model 3, but additionally adjusted for number of perceived barriers to healthy eating. A second sensitivity analysis was performed using the continuous scores for spatial access to restaurants and grocery stores as independent variables. Due to the skewed distribution of these scores, we applied squared-root transformation before adding the variables to the models. These models – called 1c, 2c and 3c are equivalent to Model 1, 2 and 3.

As we expected a dependency of observations within neighbourhoods, all models were adjusted for clustering within neighbourhoods. The estimate obtained from a multinomial logistic regression analysis is a relative risk ratio (RRR) however, to enhance interpretation, we refer to the ‘likelihood’ of cooking at home. In this way, the models indicate the likelihood of cooking at home in 4-5 and 6-7 days per week with 0-3 days per week as reference. Data analysis occurred in September 2017. The multiple imputation procedure, descriptive statistics and regression analyses were conducted with STATA 14®. Spatial analyses were conducted in ArcGIS version 10.4.

## Results

Table 1 shows the participant characteristics for the full sample and by frequency of home cooking. The mean age was 52.3 years (standard deviation (SD) 16.3) and the mean BMI was 25.2 kg/m^2^ (SD 4.5). About half of participants were female (55.5%) and highly educated (53.8%). Most participants were employed or in education (57.8%), lived in households composed by two adults without child (47.9%), and had high frequency of home cooking (6-7 days per week) (65.7%). Among those with the least access to restaurants and the least access to grocery stores, the majority (73.7% and 72.4% respectively) cooked 6-7 days per week.

**Table 1:** Participants’ characteristics by total sample and according to the frequency of cooking at home

Characteristics	Total sample		Frequency of cooking at home (days per week) *n* = 4607			
			0 – 3	4 – 5	6 – 7	
	*n*		13.1%	21.2%	65.7%	
Age - mean (SD)	5027	52.3 (16.3)	47.9 (15.6)	49.7 (15.8)	53.6 (16.3)	< 0.001^a^
Sex (%)	5025					< 0.001^b^
Male		44.5	15.5	22.0	62.6	
Female		55.5	11.2	20.7	68.2	
Educational attainment (%)	4591					0.850 ^b^
Lower		46.2	12.4	21.3	65.8	
Higher		53.8	13.4	21.2	65.3	
Household composition (%)	4593					< 0.001 ^b^
1 adult, no child		22.1	20.7	25.8	53.6	
2 adults, no child		47.9	9.75	18.9	71.3	
Adult(s), child(ren)		30.1	13.0	21.7	65.4	
Employed or in education (%)	5057					< 0.001 ^b^
No		42.2	9.59	17.8	72.7	
Yes		57.8	15.6	23.6	60.8	
BMI - mean (SD)	4503	25.2 (4.5)	25.6 (4.8)	25.3 (4.6)	25.1 (4.4)	0.006 ^a^
Number of perceived barriers to healthy eating - median (IQR)	4135	2 (0 - 4)	3 (2 - 5)	3 (1 - 4)	2 (0 - 3)	< 0.001^b^
Urban regions (%)	5076					< 0.001 ^b^
Ghent and suburbs (Belgium)		33.3	6.47	16.7	76.8	
Paris and suburbs (France)		13.9	16.4	20.4	63.2	
Budapest and suburbs (Hungary)		14.0	38.7	33.5	27.9	
The Randstad (the Netherlands)		28.5	6.04	19.5	74.5	
Greater London (UK)		10.3	12.9	23.9	63.2	
Tertiles for spatial access to restaurants	5076					< 0.001 ^b^
T1 (lowest access)		33.3	8.00	18.3	73.7	
T2		33.4	12.6	20.7	66.7	
T3 (highest access)		33.3	18.6	24.6	56.8	
Tertiles for spatial access to grocery stores	5076					< 0.001 ^b^
T1 (lowest access)		33.3	9.01	18.6	72.4	
T2		33.3	13.3	22.3	64.4	
T3 (highest access)		33.3	17.0	22.7	60.4	

^a^ ANOVA; ^b^ Chi-square; *IQR* Interquartile range

We found a tendency for an inverse association between access to restaurants and home cooking, such that participants with highest access to restaurants were less likely to report home cooking 6-7 days per week (vs. 0-3 days per week); in Model 1, individuals with the highest access to restaurants (Tertile 3 – T3) had 58% lower likelihood (95% Confidence Interval (CI) = 0.23 – 0.76; *p* = 0.004) of cooking 6-7 days per week than individuals with lowest access to restaurants (T1). For the association between spatial access to grocery stores and home cooking, we found no significant associations (Model 2). When adding the two independent variables together in the Model (Model 3), the results did not change much as compared to Model 1 (Table 2).

**Table 2:** Multinomial logistic regression analysis for access to restaurants and grocery stores with home-cooking (*n* = 5076)

		0–3/week RRR (95% CI)	4 – 5/week RRR (95% CI)	*p* value	6 – 7/week RRR (95% CI)	*p* value
Model 1						
Spatial access to restaurants	T1 (lowest)	1	1		1	
	T2		0.73 (0.49 – 1.10)	0.135	0.61 (0.35 – 1.05)	0.076
	T3 (highest)		**0.61 (0.39 – 0.96)**	0.031	**0.42 (0.23 – 0.76)**	0.004
Model 2						
Spatial access to grocery stores	T1 (lowest)	1	1		1	
	T2		0.83 (0.58 – 1.20)	0.318	0.62 (0.35 – 1.13)	0.117
	T3 (highest)		0.70 (0.47 – 1.08)	0.106	0.55 (0.29 – 1.01)	0.054
Model 3						
Spatial access to restaurants	T1 (lowest)	1	1		1	
	T2		0.75 (0.50 – 1.13)	0.171	0.63 (0.37 – 1.09)	0.101
	T3 (highest)		0.65 (0.38 – 1.12)	0.123	**0.42 (0.21 – 0.87)**	0.019
Spatial access to grocery stores	T1 (lowest)	1	1		1	
	T2		0.90 (0.65 – 1.25)	0.533	0.72 (0.43 – 1.22)	0.229
	T3 (highest)		0.90 (0.56 – 1.44)	0.652	0.91 (0.45 – 1.83)	0.783

*RRR* Relative Risk Ratio, *95%CI* 95% confidence intervals; Model 1: model with spatial access to restaurants as independent variable; Model 2: model with spatial access to grocery stores as independent variable; Model 3: model with spatial access to restaurants and spatial access to grocery stores as independent variables; T1, T2 and T3 are tertiles of spatial access, where individuals in T1 have the lowest access and individuals in T3 the highest access; All models were adjusted for age, sex, educational attainment, BMI, household composition, employment status, and urban region; Results in bold are statically significant (*p* < 0.05)

**Fig. 1:**
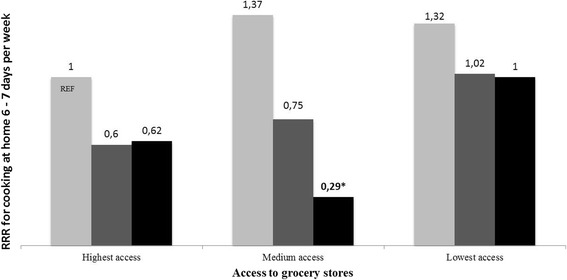
Relative Risk Ratio (RRR) as derived from multinomial logistic regression analyses indicating additive interaction between ‘spatial access to grocery stores’ and ‘spatial access to restaurants’, and cooking at home in 6-7 days per week among adults in five urban regions in Europe. The SPOTLIGHT Project (*n* = 5076). * (*p* < 0,05); REF = reference category

Table 3 and Fig. 1 present the results from the additive interaction analysis. Individuals with medium access to grocery stores (T2) and highest access to restaurants (T3) had the lowest likelihood of cooking at home 6-7 days per week, than individuals in the reference category: high access to grocery stores (T3) and low access to restaurants (T1) (RRR = 0.29; 95% CI = 0.10 - 0.84). Results for lowest and highest spatial access to grocery stores did not show a consistent tendency across tertiles of spatial access to restaurants.

**Table 3:** Multinomial logistic regression analyses with an additive interaction term between the two exposures (*n* = 5076)

**Cooking at home 0-3 days per week (Base outcome)**			
**Cooking at home 4-5 days per week - RRR (95% CI)**			
	**Spatial access to grocery stores**		
**Spatial access to restaurants**	T3 (highest access)	T2	T1 (lowest access)
T1 (lowest access)	1	1.72 (0.75 – 3.94)	1.45 (0.65 – 3.37)
T2	0.95 (0.44 – 2.08)	1.14 (0.51 – 2.53)	1.23 (0.51 – 2.94)
T3 (highest access)	0.97 (0.44 – 2.25)	0.78 (0.34 – 1.79)	1.49 (0.55 – 4.05)
**Cooking at home 6-7 days per week - RRR (95% CI)**			
	**Spatial access to grocery stores**		
**Spatial access to restaurants**	T3 (highest access)	T2	T1 (lowest access)
T1 (lowest access)	1	1.37 (0.61 – 3.06)	1.32 (0.56 – 3.12)
T2	0.60 (0.31 – 1.16)	0.75 (0.31 – 1.80)	1.02 (0.38 – 2.74)
T3(highest access)	0.62 (0.28 – 1.38)	**0.29 (0.10 – 0.84)**	1.00 (0.43 – 2.32)

*RRR* Relative Risk Ratio, *95%CI* 95% confidence intervals; The model was adjusted for age, sex, educational attainment, BMI, household composition, employment status and urban region. T1, T2 and T3 are tertiles of spatial access, where individuals in T1 have the lowest access and individuals in T3 the highest access. Results in bold was statically significant (*p* < 0.05)

Additional file 1: Table S1 shows the results of the sensitivity analysis using Model 3b (additionally adjusted for perceived barriers to healthy eating). The results from this model were very similar with those of Model 3 (Table 2), with slightly attenuated effect sizes. Additional file 1: Table S2 shows the results from sensitivity analysis using the continuous score for spatial access to restaurants and grocery stores. Models 1c, 2c and 3c were comparable with those using the tertiles of access (Table 2), the only difference was that, when using the continuous score, a significant association was found for access to grocery store and home cooking, so that a one unit increase in the squared-root score of spatial access to grocery stores decreases the likelihood of cooking at home 6-7 days per week by 0.13, as compared to home cooking 0-3 days per week (relative risk ratio 0.13; 95% confidence interval 0.02 – 0.97).

## Discussion

We explored the independent and combined associations of spatial access to grocery stores and to restaurants with frequency of home cooking in European adults. We found that a large majority of participants reported home cooking on 6-7 days per week. These participants often had low access both to restaurants and to grocery stores in their near surroundings. As we hypothesized, highest access to restaurants was associated with lower frequency of home cooking. However, we found no association for access to grocery stores. For the joint associations, we found that higher spatial access to restaurants was only associated with lower frequency of home cooking in those with medium access to grocery stores.

Comparing our findings with previous studies is difficult because they mostly focused on only one type of food retailer in relation to dietary outcomes, and none has assessed the relation with home cooking. However, a longitudinal study in the United States has examined the relation between availability of both fast food restaurants and supermarket/grocery stores with diet. Similarly to our findings, the authors found that neighbourhood availability of supermarket/grocery stores was not consistently related to diet quality, while a more consistent association was found for the relation between access to fast food restaurants and greater fast food consumption [7]. It is important to note that out-of-home food consumption is currently increasing and contributing to a larger proportion of total energy intake across all age groups [31]. As recent research shows, the use of fast-food outlets has been associated with lower overall dietary quality and higher odds of obesity [32, 33], local policy makers could therefore, consider the use of zoning and licensing to improve the mix of food retailers that do and do not sell ingredients for home-cooking.

The lack of associations between access to grocery stores and home cooking found in our study might be due to the fact that the absolute number of grocery stores in our sample was three times lower than the number of restaurants, and as a result we had lower variation in this exposure which may have led to reduced power to detect associations for grocery stores. A higher number of retailers that mostly sell food to be consumed away from home, in our case represented by restaurants, rather than grocery stores was also found in other studies [34, 35]. Another explanation may be that grocery stores sell a wide range of products [36] which include both raw ingredients to cook at home as well as take away meals. Therefore, the presence of a supermarket in the neighbourhood may enable but at the same time discourage the preparation of meals at home. Considering that food retailers such as full-service restaurants and fast food restaurants sell prepared meals, the consistent inverse association between this type of stores and frequency of home cooking found in this study is plausible. Nonetheless, the findings should be interpreted with caution because although a high availability of restaurants in a neighbourhood may discourage home cooking, the presence of full-service restaurants may be more favourable for diet quality than the presence of fast food restaurants [37, 38]. A third explanation may be that we examined spatial access to grocery stores and to restaurants in the surroundings of our participants’ home, while participants may do their grocery shopping outside their residential area, or in a part of their neighbourhood that is not covered by using the administrative boundaries [39]. In that respect, it could be that access to grocery stores in the residential neighbourhood may be less important for frequency of home cooking, and that more general accessibility measures, for instance those related to travel time and affordability may be more important [40, 41]. Studies on time use may be useful in order to examine where and when people do their grocery shopping, and other areas than the home food environment should be examined for their influence on cooking practices, such as the work and leisure environment, as well as commuting routes.

Previous literature on home cooking identified important individual-level determinants of cooking behaviour such as sex, time availability and employment status, cultural background, ethnicity, attitude and self-efficacy [16]. Although we did not have information about ethnic background, type of occupation and age of any children in the household, it is a strength of this study that we considered many individual-level determinants as covariates in the assessment of food environmental correlates of home cooking. Future studies could further zoom into the interactions of individual-level with environment-level determinants of home cooking, for instance by investigating whether the association between the food environment and home cooking is different according to household composition or education. Additional strengths of this study include that we accounted for different types of exposures to the food environment by analysing the independent and combined effects of grocery stores and restaurants on home cooking, and our results are based on a large sample from different European countries. Some limitations also need to be acknowledged. First, like most population-based studies [42], the low response rate could have led to selection bias, possibly reducing the external validity of our study [22]. In addition, our study focused on the residential neighbourhood food environment, and did not include potentially important food environment information from non-residential exposure settings such as work or leisure food environments [43]. Cooking practices may be associated with ethnic or cultural background. However, ethnicity could not be included in the survey questionnaire in two of the participating countries (due to ethical restrictions), and ethnicity was recorded differently in the other three. Therefore, ethnicity could not be included in the analyses. Finally, our cross-sectional design does not allow for a distinction between a causal association of spatial access to restaurants with home cooking and potential selection effects (e.g., it is unknown whether the opening of new restaurants reflects the demand of the residents, or individuals who do not like cooking choose to live in neighbourhoods that have many restaurants available).

## Conclusions

Greater spatial access to restaurants was associated with lower likelihood of frequent home cooking, while no association between access to grocery stores and home cooking was found. We did not find strong evidence for a joint association of spatial access to grocery stores and spatial access to restaurants with home cooking. In general, access to restaurants showed to be relevant, largely independent of access to neighbourhood grocery stores. The outcomes of this pioneering study might serve as a base for future studies on the upstream determinants of home cooking. Further research should seek to refine measures of access to the food environment in relation to home cooking, for instance by looking at different exposure settings such as the work environment and by focusing on individuals with higher spatial access to restaurants.

## Additional file

Additional file 1: Sensitivity analyses. (DOCX 20 kb)

## Abbreviations

95%CI: 95% confidence interval
IQR: Interquartile range
RERI: Relative excess risk due to interaction
RRR: Relative risk ratio
SD: standard deviation
SPOTLIGHT-VAT: SPOTLIGHT virtual audit tool
T1: Tertile 1
T2: Tertile 2
T3: Tertile 3
