# Supplementary material for: Spatial access to restaurants and grocery stores in relation to frequency of home cooking
Source: Int J Behav Nutr Phys Act. 2018 Jan 16;15:6. doi: 10.1186/s12966-017-0640-6 (PMC5771126; doi:10.1186/s12966-017-0640-6)
Supplement: Additional file 1: — Sensitivity analyses. (DOCX 20 kb) [file 12966_2017_640_MOESM1_ESM.docx]

**Supplementary tables**

**Supplementary Table 1.** Relative Risk Ratio (RRR) and 95% confidence intervals (95%CI) as sensitivity analysis derived from multinomial logistic regression analyses indicating the associations between spatial access to restaurants and grocery stores with weekly frequency of cooking at home additionally adjusted for number of perceived barriers to healthy eating. The SPOTLIGHT Project (n=5076).

|  |  | 0 – 3/week  RRR (95% CI) | 4 – 5/week  RRR (95% CI) | p value | 6 – 7/week  RRR (95% CI) | p value |
| --- | --- | --- | --- | --- | --- | --- |
| **Model 3b** | |  |  |  |  |  |
| **Spatial access to restaurants** | T1 (lowest) | 1 | 1 |  | 1 |  |
|  | T2 |  | 0.77 (0.51 – 1.16) | 0.210 | 0.68 (0.40 – 1.14) | 0.148 |
|  | T3 (highest) |  | 0.68 (0.68 – 1.19) | 0.167 | **0.49 (0.24 – 1.00)** | 0.049 |
| **Spatial access to grocery stores** | T1 (lowest) | 1 | 1 |  | 1 |  |
|  | T2 |  | 0.94 (0.69 – 1.27) | 0.666 | 0.78 (0.49 – 1.27) | 0.319 |
|  | T3 (highest) |  | 0.93 (0.59 – 1.48) | 0.765 | 0.98 (0.51 – 1.89) | 0.954 |

Model 3b adjusted for age, sex, educational attainment, BMI, household composition, employment status, urban region and perceived barriers to healthy eating; T1, T2 and T3 are tertiles of spatial access, where individuals in T1 have the lowest access and individuals in T3 the highest access; Results in bold are statically significant (p<0.05).

**Supplementary Table 2**. Relative Risk Ratio (RRR) and 95% confidence intervals (95%CI) as sensitivity analysis derived from multinomial logistic regression analyses indicating the associations between spatial access to restaurants and grocery stores with weekly frequency of cooking at home using the continuous score for spatial access as intendent variable. The SPOTLIGHT Project (n=5076).

|  |  | 0 – 3/week  RRR (95% CI) | 4 – 5/week  RRR (95% CI) | p value | 6 – 7/week  RRR (95% CI) | p value |
| --- | --- | --- | --- | --- | --- | --- |
| **Model 1c** | |  |  |  |  |  |
| **Spatial access to restaurants** |  | 1 | **0.38 (0.15 – 0.97)** | 0.042 | **0.22 (0.05 – 0.91)** | 0.036 |
| **Model 2c** | |  |  |  |  |  |
| **Spatial access to grocery stores** |  | 1 | 0.30 (0.07 – 1.17) | 0.083 | **0.13 (0.02 – 0.97)** | 0.046 |
| **Model 3c** | |  |  |  |  |  |
| **Spatial access to restaurants** |  | 1 | 0.36 (0.86 – 1.48) | 0.155 | 0.24 (0.02 – 2.38) | 0.222 |
| **Spatial access to grocery stores** |  | 1 | 1.14 (0.17 – 7.76) | 0.893 | 0.85 (0.04 – 2.38) | 0.917 |

All models were adjusted for age, sex, educational attainment, BMI, household composition, employment status, urban region and perceived barriers to healthy eating; T1, T2 and T3 are tertiles of spatial access, where individuals in T1 have the lowest access and individuals in T3 the highest access; Results in bold are statically significant (p<0.05).
